# Experience with the Urinary Tetrasaccharide Metabolite for Pompe Disease in the Diagnostic Laboratory

**DOI:** 10.3390/metabo11070446

**Published:** 2021-07-08

**Authors:** Jennifer T. Saville, Maria Fuller

**Affiliations:** 1 Genetics and Molecular Pathology, SA Pathology at Women’s and Children’s Hospital, 72 King William Road, North Adelaide, SA 5006, Australia; jennifer.saville@adelaide.edu.au; 2 Adelaide Medical School, University of Adelaide, North Terrace, Adelaide, SA 5000, Australia

**Keywords:** Pompe disease, diagnosis, glycogen, lysosomal storage disorder, urinary tetrasaccharide, biomarker, biochemical monitoring, glycogen storage disease, mass spectrometry

## Abstract

Following clinical indications, the laboratory diagnosis of the inherited metabolic myopathy, Pompe disease (PD), typically begins with demonstrating a reduction in acid alpha-glucosidase (GAA), the enzyme required for lysosomal glycogen degradation. Although simple in concept, a major challenge is defining reference intervals, as even carriers can have reduced GAA, and pseudodeficiencies complicate interpretation. Here, we developed a mass spectrometric assay for quantification of a urinary glycogen metabolite (tetrasaccharide) and reported on its utility as a confirmatory test for PD in a diagnostic laboratory. Using two age-related reference intervals, eight returned tetrasaccharide concentrations above the calculated reference interval but did not have PD, highlighting non-specificity. However, retrospective analysis revealed elevated tetrasaccharide in seven infantile-onset (IOPD) cases and sixteen late-onset (LOPD) cases, and normal concentrations in one heterozygote. Prospective tetrasaccharide analysis in nine individuals with reduced GAA confirmed IOPD in one, LOPD in six and identified two heterozygotes. Using this metabolite as a biomarker of therapeutic response was not overly informative; although most patients showed an initial drop following therapy initiation, tetrasaccharide concentrations fluctuated considerably and remained above reference intervals in all patients. While useful as a confirmation of PD, its utility as a biomarker for monitoring treatment warrants further investigation.

## 1. Introduction

Pompe disease (PD) is an inherited, progressive, metabolic myopathy resulting from the lysosomal accumulation of glycogen due to a deficiency in the lysosomal hydrolase, acid alpha-glucosidase (GAA; EC 3.2.1.20). The disease is broadly divided into two forms. Infantile-onset PD (IOPD) typically presents within the first six months of life with cardiomyopathy, respiratory dysfunction and hypotonia and, if left untreated, premature death results from cardiorespiratory failure [1,2]. Late-onset PD (LOPD) manifests later in life, with limb and respiratory muscle weakness and a slower progressing disease course [3]. The clinical manifestations of LOPD are highly varied, as is age of symptom onset, and they are often confused with more common muscular dystrophies.

Early diagnosis of IOPD is critical for life-saving treatment in the form of enzyme replacement therapy (ERT) that reduces ventilation dependence and mortality risks by up to 99% [4]. Likewise, in LOPD, the mortality rate reportedly decreased five-fold in treated patients compared to those who were untreated [5]. Confirming a suspected diagnosis of PD makes use of the biochemical and genetic characteristics of the disease. Initial testing, just like that used in PD newborn screening programs [6], measures GAA activity in dried blood spots (DBS) using artificial substrates and detection, either with fluorescence [7] or mass spectrometry [8,9]. Reduced GAA activity in DBS is reported in both IOPD and LOPD patients [10], but second tier testing is required to confirm a diagnosis due to pseudodeficiency alleles which result in a reduction in GAA activity in vitro but no active disease [11]. Molecular screening for *GAA* genetic variants can be used as confirmation of PD but is complicated by a high number of genetic variants of uncertain significance (VUS) [12,13] that still require phenotype confirmation.

Another biochemical feature of PD is the accumulation of lysosomal glycogen; however, this is restricted to biopsied muscle tissue and measurement is not straightforward [14]. Amylolytic degradation of glycogen, followed by α-1,4 glucosidase activity, produces a tetrasaccharide, 6-α-D-glucopyranosyl-maltotriose, a metabolite of glycogen that is excreted in the urine [15]. Present in all urine, this tetrasaccharide has been shown to be elevated in patients with glycogen storage disorders, including Pompe disease [16,17,18,19]. Several studies have shown the urine tetrasaccharide is elevated in patients with IOPD and LOPD [20,21] but, importantly, not in individuals carrying the pseudodeficiency allele [18,22]. The urine tetrasaccharide has also shown promise as a biomarker for monitoring patients post initiation of ERT [23,24,25]. In particular, Young et al. [24] found urine tetrasaccharide concentrations correlated with muscle glycogen content at 52 weeks post-ERT, and that patients with higher pre-treatment tetrasaccharide concentrations had poorer motor function outcomes. Here, we describe the development and validation of a mass spectrometric method for the measurement of the tetrasaccharide in our diagnostic service laboratory, and thereafter report on the outcome of this urinary metabolite for the diagnosis of PD, as well as for biochemical monitoring of patients in receipt of ERT.

## 2. Results

### 2.1. Urine Tetrasaccharide Method Validation

**Figure 1:**
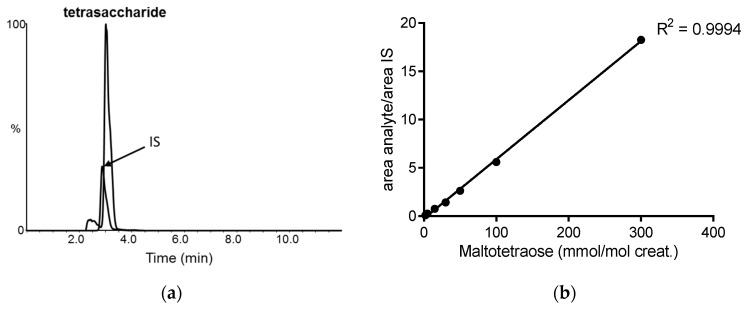
Liquid chromatography tandem mass spectrometry of urine tetrasaccharide: (**a**) extracted ion chromatogram of the tetrasaccharide and internal standard (IS); (**b**) calibration curve of maltotetraose concentration plotted against the ratio of the peak area of the analyte to that of the IS.

The internal standard (IS) and tetrasaccharide eluted at the same retention time (2.9 min) on the pentafluorophenyl (PFP) liquid chromatography (LC) column with no interfering peaks (Figure 1a), with maltotetraose also eluting at 2.9 min. The calibration curve was linear across the concentration range of 2–300 mmol/mol creatinine (Figure 1b). The limit of detection was calculated as three times the signal-to-noise at 0.02 mmol/mol creatinine, whereas the limit of quantification was 0.08 mmol/mol creatinine, at which the target concentration was achieved within a 10% margin of error. The interassay CV was 9%, determined using a PD patient sample for 65 separate assays over a twelve-month period. Recovery was 82% (*n* = 8), determined by measurement of maltotetraose (120 mmol/mol creatinine) from control urine. The tetrasaccharide was stable in urine after three cycles of freeze/thaw, stored at room temperature for up to one week and at −20 °C for up to 17 months (data not shown). Prepared derivatized urine samples were stable for up to 36 h in the autosampler at 18 °C. Of note, preparation of quality control (QC) material by the addition of maltotetraose to control urine was not stable, with an observed reduction in the calculated concentration following storage at −20 °C and −70 °C (data not shown). To overcome this, we employed PD patient urine as low (8 mmol/mol creatinine) and high (40 mmol/mol creatinine) QC material.

**Figure 2:**
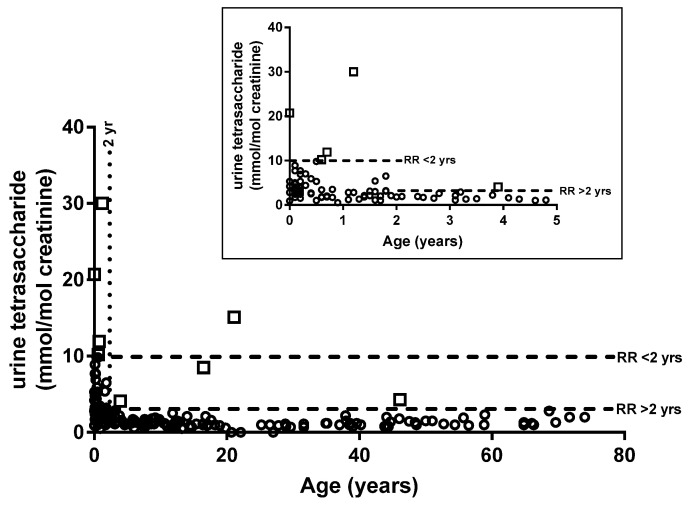
Age-related reference intervals. Urine tetrasaccharide concentrations for 190 individuals without Pompe disease. Inset shows expanded results for individuals < 5 years old. White squares indicate individuals with concentrations above the reference interval. RR, reference range.

Urine tetrasaccharide concentrations from 190 patients without PD were used to calculate reference intervals using a 95% percentile upper limit. Figure 2 shows that tetrasaccharide concentrations were higher in individuals < 2 years of age and, as such, two age-related reference ranges were determined (1) <10 mmol/mol creatinine for patients < 2 years of age (*n* = 67) and (2) <3 mmol/mol creatinine for patients > 2 years old (*n* = 123). There were eight patients (4 patients < 2 and 4 > 2 years of age) who returned urine tetrasaccharide concentrations above the calculated reference intervals but did not have PD (Figure 2).

### 2.2. Diagnosis of PD

Archived DBS and spot urine samples were available from 23 genetically confirmed cases of PD. Seven patients with IOPD returned reduced GAA activities (0.0–0.9 pmol/spot/h), which were well below the normal reference range of 2.9–23.4 pmol/spot/h (Table 1). Total α-glucosidase activity is included in the GAA assay protocol as a measure of sample integrity, with an elevated total α-glucosidase/GAA ratio also providing an additional indicator of PD, ranging from 32 to 482 for the seven IOPD patients (reference range 2.5–14.6). Urine tetrasaccharide concentrations were also clearly elevated above the reference range for all IOPD patients (Table 1). Sixteen confirmed LOPD patients were demarcated from controls by reduced GAA activity and elevated urine tetrasaccharide concentrations (Table 1). Another patient, heterozygous for the c.-32-13T>G variant had a GAA activity of 1.7 pmol/spot/h and a neutral/GAA ratio of 18, but the urinary tetrasaccharide concentration was within the normal range.

Since the introduction of the urine tetrasaccharide mass spectrometry assay within diagnostic service, seven prospective diagnoses of PD patients have been reported in the two-year period (Table 1). This included one 3-month-old presenting with cardiomyopathy and diagnosed with IOPD, and a remaining six patients diagnosed with LOPD. Two patients with low GAA but normal urine tetrasaccharide were confirmed heterozygotes by molecular testing, and a further five were shown to have reduced GAA activity (0.5–2.5 pmol/spot/h). The latter returned normal urine tetrasaccharide concentrations with no further follow-up because the diagnosis of PD was no longer considered.

**Figure 3:**
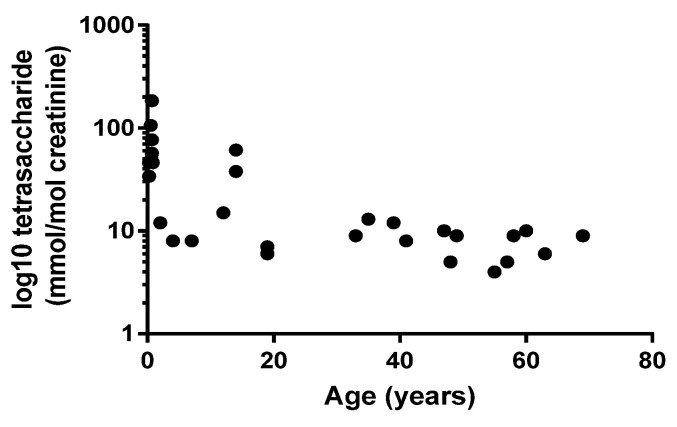
Relationship between patient age at diagnosis and urine tetrasaccharide concentrations. Pearson’s *r* = −0.543; *p* = 0.002.

Figure 3 shows that there was an inverse relationship between age at diagnosis and urine tetrasaccharide concentration (Pearson’s *r* = −0.543; *p* < 0.01), but no correlation was observed between age of onset and GAA activity (Pearson’s *r* = 0.358; *p* = 0.05) or between GAA activity and urine tetrasaccharide concentration (Pearson’s *r* = −0.011; *p* = 0.59).

### 2.3. Longitudinal Monitoring of PD Patients

**Figure 4:**
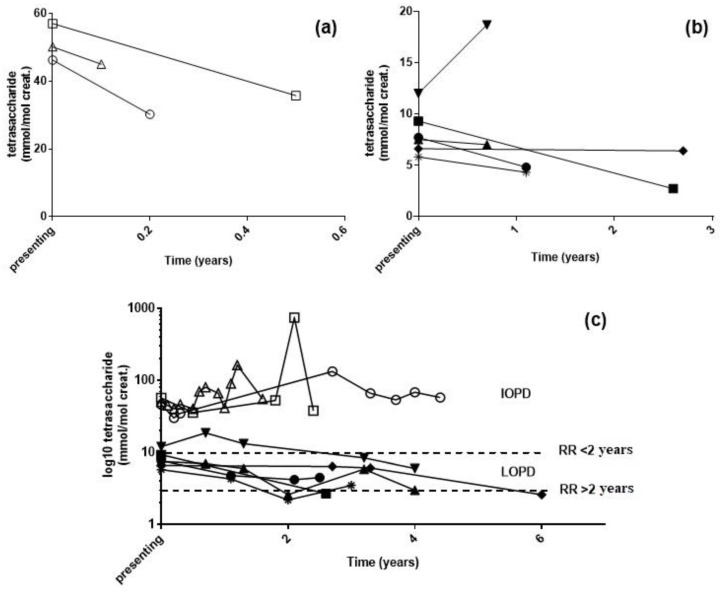
Urine tetrasaccharide concentrations following enzyme replacement therapy: (**a**) IOPD and (**b**) LOPD patients at diagnosis and first urine post-treatment. (**c**) Long-term monitoring of patients following treatment. RR, reference range. △ c.953T>C homozygous, 0.6 years at diagnosis; ◯ c.323G>A homozygous, 0.9 years at diagnosis; ☐ c.266G>T/c.2815_2816del, 0.7 years at diagnosis; ▲ c.[-32-13T>G; 2275G>A]/c.1827delC, 4 years at diagnosis; ▼ c.[-32-13T>G; 2275G>A]/c.1827delC, 2 years at diagnosis; ◆ c.2297A>C/c.-32-13T>G, 11 years at diagnosis; ● c.-32-13T>G/c.1735G>A, 8 years at diagnosis; ■ c.-32-13T>G/c.1827delC, 58 years at diagnosis; 🞽 c.-32-13T>G/c.1548G>A, 17 years at diagnosis.

For nine PD patients, urine samples were available pre- and post-ERT (Figure 4). Three patients, diagnosed with IOPD between 7 and 11 months of age, continued to have elevated urinary tetrasaccharide concentrations following ERT, despite an initial drop in concentration in the first urine collected post-treatment. For the remaining six LOPD patients, urine tetrasaccharide concentrations were variable in response to treatment. Only one patient was an adult at the time of diagnosis (58 years of age), and the urine tetrasaccharide concentration was the second highest for LOPD patients at time of diagnosis (9 mmol/mol creatinine) but dropped to normal 2.5 years after treatment. A second patient, diagnosed at 11 years, continued to have elevated tetrasaccharide concentrations up to 3 years post-treatment; these reduced to within normal limits following 6 years of ERT. The youngest LOPD patient at diagnosis was 1.9 years of age, who was diagnosed due to an older sibling. Both were heterozygous for three pathogenic variants c.[-32-13T>G; 2275G>A(p.Gly759Arg)]/c.1827delC(p.Tyr609*) and had the highest urine tetrasaccharide concentration at diagnosis (12 mmol/mol creatinine) that remained elevated after 4 years of ERT. The older sibling (3.8 years at diagnosis) had variable urine tetrasaccharide concentrations post-treatment, which reduced to normal at 5.8 years of age before increasing to 6 mmol/mol by 7 years. Similarly, a patient diagnosed at 17.5 years of age returned normal tetrasaccharide concentrations 2 years post-ERT before increasing to 4 mmol/mol creatinine the following year. The final patient, diagnosed at 7.6 years of age, continued to have elevated urine tetrasaccharide up to 3 years post-ERT.

## 3. Discussion

To address the need for secondary confirmation of reduced GAA for diagnosing PD, we developed a simple assay to quantify the amount of a tetrasaccharide in urine, which is presumed to be a metabolite of glycogen. This tetrasaccharide is excreted at higher concentrations in patients with PD compared with individuals harboring the pseudodeficiency, who have no glycogen accumulation [22]. Here, we show that measuring the urine tetrasaccharide following low blood GAA activity is a fast and informative method for the diagnosis of IOPD and LOPD. The use of derivatizing reagents shortens chromatographic run times and, although our run time at 13 min is longer than the recent 5 min reported using butyl 4-aminobenzoate [19], we do not require solid-phase extraction to remove 1-phenyl-3-methyl-5-pyrazolone (PMP), significantly simplifying sample preparation.

Our results concur with previous methods reporting urinary tetrasaccharide elevations in IOPD and LOPD patients compared with control subjects. However, we made use of just two reference intervals for control subjects, <2 years of age of 10 mmol/mol creatinine and <3 mmol/mol creatinine for the cohort aged over two. Previous methods, using the derivatizing reagent butyl 4-aminobenzoate [20] and without derivatization [17], required five sets of reference intervals from <1 to >20 years of age. Using just two reference intervals, the diagnostic sensitivity of our method was 100%, while specificity was 96%; these results are similar to, although slightly improve upon, the 98.5% sensitivity and 92% specificity previously reported [17]. Elevations in urinary tetrasaccharide in individuals without PD are not unexpected given that this metabolite is not specific to PD and elevations have also been observed in other glycogen storage diseases [17], Duchenne muscular dystrophy [26] and in patients with acute pancreatitis, muscular trauma, urinary tract infections and some cancers [27]. Kumlien et al. [27] also determined that a high carbohydrate diet could elevate the urine tetrasaccharide up to three-fold. In this study, one neonate who returned a tetrasaccharide concentration of 21 mmol/mol creatinine was hypoglycemic, with standard treatment involving glucose supplementation. Among the other seven individuals that returned urine tetrasaccharide concentrations above the reference interval, four were only marginally elevated. Two individuals aged 6 and 8 months returned a concentration of 10 and 12 mmol/mol creatinine, respectively, whereas a 4 year old and a 46 year old both had tetrasaccharide concentrations of 4 mmol/mol creatinine. Given these outliers, we assessed whether more defined age-related reference intervals would have normalized these samples, noting that other studies had five groups [17,19,20]. Using a 90% upper percentile (required due to smaller sample numbers in each group), we calculated 11 false positives over the age ranges reported by both An et al. [20] and Sluiter et al. [17], and 16 false positives over the five age ranges reported by Piraud et al. [19]. Using only two age ranges, therefore, allowed a greater specificity while still enabling 100% sensitivity.

We note that the method described here cannot distinguish between isomers; while, in glycogen storage diseases, including PD, this tetrasaccharide is commonly referred to as a glucose tetrasaccharide [27], this is an assumption only. While the mass spectrometry method measures a mass to charge ratio which could include any four hexose moieties, An et al. [20] used high-performance liquid chromatographic separation to show that PD patient urine predominantly contained glucose tetrasaccharide with no appreciable amounts of the isomer maltotetraose. Previous studies support the hypothesis that tetrasaccharide is derived from muscle glycogen by showing a statistically significant correlation between tetrasaccharide concentration and glycogen storage in the muscles of PD patients [28,29]. In contrast to An et al., Sluiter et al. [17] reported that maltotetraose was present in approximately 5% of urine samples, predominantly in newborns. In our study, 27 control urine samples were tested from infants younger than 3 months of age, and only one had elevated urine tetrasaccharide concentrations, which could be explained by glucose supplementation.

**Figure 5:**
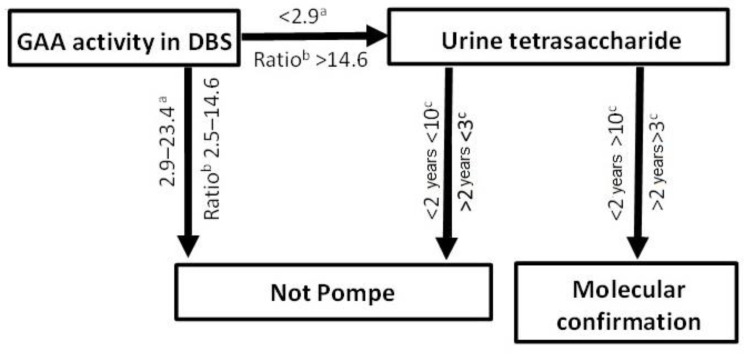
Laboratory diagnostic pathway for Pompe disease. ^a^ pmol/spot/h; ^b^ ratio of total α-glucosidase/GAA; ^c^ mmol/mol creatinine.

The primary role of the urinary tetrasaccharide resides in the diagnostic trajectory of PD to validate low GAA determinations (Figure 5). Although no individuals with the pseudodeficiency were identified in our cohort, three individuals aged 1, 27 and 69 years of age, with GAA below the reference interval, were carriers of PD variants; the normal urinary tetrasaccharide virtually excluded a PD diagnosis, highlighting its utility (Table 1). Therefore, if the urinary tetrasaccharide is within the normal range, a diagnosis of PD may be dismissed, thus avoiding unnecessary molecular testing and allowing other avenues of investigation to be pursued to explain symptomology.

Although classic IOPD, which presents within the first three months of life, has prototypical clinical presentations that rarely go unrecognized, there is a broad spectrum of phenotypes which is influenced by a number of factors, including residual enzyme activity. Residual enzyme activity is typically < 3% of the mean of normal in classic IOPD, but in non-classical/juvenile OPD and LOPD, residual activity is 3–30% [30,31], meaning GAA is a poor predictor of disease severity. In our cohort of PD patients, we also showed that there was no correlation between age at diagnosis and GAA activity, but we did show a correlation with urine tetrasaccharide concentrations. Figure 3 shows that higher urine tetrasaccharide concentrations were associated with younger patients, and that older patients (LOPD) had lower concentrations. Our data therefore support previous findings reporting correlations with urinary tetrasaccharide, age of symptom onset and PD phenotype [19,29].

Inadequate monitoring and clinical data were available from our small set of PD patients on ERT to fully assess the utility of the urine tetrasaccharide as a response biomarker. Longitudinal monitoring of PD patients post-ERT revealed that urinary tetrasaccharide concentrations did not normalize in all patients. In particular, IOPD patients, despite showing an initial drop, still had significant urinary tetrasaccharide concentrations up to four years following ERT. Chien et al. [32] recently reported correlations between urinary tetrasaccharide and ERT outcomes in patients with IOPD, with ambulate patients having significantly lower urinary tetrasaccharide concentrations compared with patients unable to walk. Of note, they found that all patients sustained concentrations above the normal reference range, in agreement with our data. Little data are currently available on ERT outcomes in patients with LOPD or on urinary tetrasaccharide concentrations, and our data suggest that there is a general trend of reducing concentrations following the initiation of therapy (Figure 4).

## 4. Materials and Methods

### 4.1. Materials

All solvents were purchased from Merck (Darmstadt, Germany). NH_4_OH (30% *v*/*v*), HCOOH (98% *v*/*v*) and CHCl_3_ (containing 1% CH_3_CH_2_OH) were reagent grade, and CH_3_OH, H_2_O, (CH_3_)_2_CHOH and CH_3_CN LiChroSolv were gradient grade. NH_4_COOH (HPLC grade) and chondroitin disaccharide Δdi-0S were purchased from Sigma Aldrich (St. Louis, MO, USA). 1-phenyl-3-methyl-5-pyrazolone (PMP) was purchased from Tokyo Chemical Industry Co., Ltd. (Tokyo, Japan). Maltotetraose was purchased from Carbosynth Ltd. (Crompton, Witham, UK).

### 4.2. Patient Samples

All DBS and urine samples were submitted to our department for the diagnosis and biochemical monitoring of metabolic diseases. The use of patient samples in this study was approved by the Institutional Human Research Ethics Committee (HREC/15/WCHN/69). All DBS were stored at room temperature and urine samples were stored at −20 °C and thawed at room temperature prior to analysis.

### 4.3. Urinary Tetrasaccharide Determination

A seven-point standard curve was prepared with each assay by addition of maltotetraose in 0.01 mL of H_2_O to give final concentrations of 2, 5, 15, 30, 50, 100, 300 mmol/mol creatinine. Patient urine (0.05 µmol creatinine equivalent) and standards were lyophilized before reconstitution with 1 nmol of chondroitin disaccharide Δdi-0S (IS) in 0.1 mL of PMP (0.25 M in 0.4 M of NH_4_OH; pH 9.5). Samples were incubated at 70 °C for 90 min prior to acidification with 0.5 mL of 0.2 M HCOOH. Excess PMP was removed by shaking with CHCl_3_ (0.5 mL) for 1 min prior to centrifugation for 5 min (13,000× *g*; RT). The organic phase was removed and the wash step was repeated a further three times. The aqueous phase (0.1 mL) was removed and placed in the well of a 96-well microtiter plate, which was heat-sealed for analysis by liquid chromatography coupled with electrospray ionization tandem mass spectrometry (LC-ESI-MS/MS).

Desalting and partial separation of samples was achieved on a Pursuit 3 pentafluorophenyl (PFP) column (2.0 × 100 mm; 3 µm; Agilent Technologies, Santa Clara, CA, USA) maintained at 30 °C with an Agilent Affinity 1290 inline filter containing a 0.3 µm of frit placed in front of the column. Samples were maintained at 18 °C in the autosampler with 2 µL injected into the mobile phase, which was at a flow rate of 0.3 mL/min. Mobile phase A consisted of H_2_O with 0.1% HCOOH and mobile phase B contained CH_3_CN with 0.1% HCOOH. The column was equilibrated at 20% mobile phase B with a linear ramp to 22.5% mobile phase B over 5 min, followed by an increase to 90% mobile phase B at 5.5 min. This was maintained for 2 min before being decreased to 20% mobile phase B at 8 min, where the column was re-equilibrated for 4 min prior to injection of the next sample. The first 2.1 min was sent to waste before being directed into the ESI source (ES 5000 V) of an AB SCIEX QTRAP 6500 tandem mass spectrometer in positive ion mode and with an ion source temperature of 300 °C. Nitrogen was used for curtain gas, 25 units; collision gas, high; nebulizer gas 1, 20 units and auxiliary gas 2, 40 units. Analytes were measured by multiple reaction monitoring with the transitions 997/175 and 710/253 for the tetrasaccharide and IS, respectively.

Urinary tetrasaccharide concentrations were determined using a calibration curve created in MultiQuant (SCIEX; v. 3.0.1) of the maltotetraose concentration and the ratio of chromatographic peak area of maltotetraose and the IS, using a 1/x^2^ weighting. Acceptance for the calibration curve was a coefficient of determination *r*^2^ ≥ 0.99, and each standard had to be within 15% of the target concentration. Tetrasaccharide concentration was normalized to creatinine concentration and expressed as mmol/mol creatinine.

### 4.4. GAA Activity in Dried Blood Spots

GAA activity was measured from 3 mm DBS, as previously described [7]. In brief, blood was eluted from the DBS and α-glucosidase measured using the fluorometric substrate 4-methylumbelliferyl-α—D-glucopyranoside. Acarbose was added under acidic conditions to inhibit maltase-glucoamylase and allow for the measurement of GAA activity by release of the 4-methylumbelliferyl (4-MU) moiety. Total neutral α-glucosidase (pH 7.0) was used as a measure of sample integrity. A calibration curve consisting of 4-MU (0–1 nM) was used to calculate enzyme activity following background subtraction to account for autofluorescence.

## 5. Conclusions

Following initial enzymatic investigations suggestive of PD, the urinary tetrasaccharide is a useful metabolite in a second-tier laboratory test to confirm or exclude a diagnosis of PD. This report provides additional and much needed data, supporting the utility of the urinary tetrasaccharide in the diagnostic setting of PD, especially given that there are very few literature reports on the measurement of this metabolite. As a biomarker to monitor treatment response in PD patients, further work is clearly needed, although our data does show that the tetrasaccharide responds, at least to some extent, to ERT.

## Figures and Tables

**Table 1:** GAA activity, urine tetrasaccharide concentration and genotype of patients previously confirmed with Pompe disease, as well as those diagnosed since the initiation of the urine tetrasaccharide assay within the diagnostic laboratory.

	Group	Age (years)	GAA Activity (pmol/spot/h) ^a^	Ratio Total/GAA Activity ^b^	Urine Tetrasaccharide (mmol/mol creatinine) ^c^	Genotype
Confirmed	IOPD	0.6	0.0	482	50	c.953T>C/c.953T>C
	IOPD	0.7	0.9	32	57	c.266G>T/c.2815_2816del
	IOPD	0.7	0.7	89	77	Genotype not known ^†^
	IOPD	0.25	0.9	59	46	Genotype not known ^†^
	IOPD	0.8	0.3	142	46	c.323C>A homozygous
	IOPD	0.7	0.8	62	185	c.[2155G>C; 1726C>A; 2065G>A] homozygous
	IOPD	0.5	0.7	65	106	Genotype not known
	LOPD	47	0.9	57	10	c.-32-13T>G/c.1827del
	LOPD	69	0.7	39	9	c.-32-13T>G/c.953T>C
	LOPD	7	0.0	4417	8	c.-32-13T>G/c.1735G>A
	LOPD	41	0.8	31	8	c.-32-13T>G/c.307T>G
	LOPD	4	0.2	260	8	c.1827del/c.[-32-13T>G; 2275G>A] ^d^
	LOPD	14	0.3	101	61	Genotype not known ^e^
	LOPD	14	0.6	95	38	Genotype not known ^e^
	LOPD	2	0.5	88	12	c.1827del/c.[-32-13T>G; 2275G>A] ^d^
	LOPD	19	0.5	116	7	c.-32-13T>G/c.307T>G
	LOPD	58	0.9	51	9	c.-32-13T>G/c.1827del
	LOPD	12	0.4	69	15	Genotype not known
	LOPD	19	0.4	132	6	c.-32-13T>G/c.482_483del
	LOPD	35	0.5	61	13	c.-32-13T>G/c.482_483del
	LOPD	39	0.6	34	12	Genotype not known
	LOPD	19	0.8	37	6	Genotype not known
	LOPD	60	1.0	43	10	c.-32-13T>G/c.1441T>C
	Het	69	1.7	18	1	c.-32-13T>G
Diagnosed	IOPD	0.25	0.2	200	34	Genotype not known ^†^
	LOPD	33	1.1	56	9	Genotype not known
	LOPD	48	0.2	149	5	Genotype not known
	LOPD	57	0.9	84	5	c.-32-13T>G/c.1075+1G>T
	LOPD	49	1.9	52	9	c.-32-13T>G/c.2481+109_2646+38del
	LOPD	63	0.2	246	6	Genotype not known
	LOPD	55	0.7	113	4	Genotype not known
	Het	1	2.7	14	1	c.-32-13T>G
	Het	27	2.4	13	1	c.2481+109_2646+38del

^a^ Reference range (RR) 2.9–23.4 pmol/spot/h; ^b^ RR 2.5–14.6; ^c^ RR < 2 years < 10, >2 years < 3 mmol/mol creatinine; ^d,e^ siblings; Het, heterozygote; ^†^ cardiomyopathy at presentation. All results measured at diagnosis.

## Data Availability

Data is contained within the article.
